# A46 ENTEROBACTERIACEAE ENRICHMENT IN THE GUT MICROBIOME OF CRITICALLY ILL PATIENTS PROMOTES IMMUNE DYSFUNCTION AND SUSCEPTIBILITY TO NOSOCOMIAL INFECTIONS

**DOI:** 10.1093/jcag/gwac036.046

**Published:** 2023-03-07

**Authors:** J M Schlechte, A Z Zucoloto, I -L Yu, C J Doig, M J Dunbar, K D McCoy, B McDonald

**Affiliations:** 1 Department of Critical Care Medicine; 2 Snyder Institute for Chronic Diseases; 3 Department of Physiology and Pharmacology; 4 Department of Community Health Sciences; 5 Alberta Children’s Hospital Research Institute; 6 Department of Pediatrics, University of Calgary, Calgary, Canada

## Abstract

NOT PUBLISHED AT AUTHOR’S REQUEST

**Please acknowledge all funding agencies by checking the applicable boxes below:**

CIHR, Other

**Please indicate your source of funding;:**

Government of Alberta

**Disclosure of Interest:**

None Declared

